# Functional blocking of Varicella Zoster Virus prevents acquisition of Alzheimer’s disease‐like characteristics induced by reactivation of quiescent Herpes Simplex Virus Type 1

**DOI:** 10.1002/alz.090592

**Published:** 2025-01-09

**Authors:** Dana M Cairns, Ruth F Itzhaki, David L Kaplan

**Affiliations:** ^1^ Tufts University, Medford, MA USA; ^2^ The University of Manchester, Manchester United Kingdom; ^3^ University of Oxford, Oxford United Kingdom

## Abstract

**Background:**

Hallmark features of AD are well defined, however, the generation of in vitro models of sporadic AD poses a significant challenge due to the complex, undefined etiology and slow progression of this disease. Herpes simplex virus type I (HSV‐1) is a pathogen that is gaining increasing attention as a potential causative agent in AD pathogenesis. HSV‐1 is a DNA virus that typically resides throughout the peripheral nervous system in a latent state. We have previously shown that HSV‐1 infection of human induced neural stem cells (hiNSCs) robustly mimic human disease with amyloid plaque–like formations, P‐tau accumulation, reactive gliosis, neuroinflammation, and diminished neural network functionality; completely in the absence of any exogenous mediators of AD. Further, we have demonstrated that quiescent HSV‐1 can be reactivated by various mechanisms, which also leads to AD‐like phenotypes. Varicella zoster virus (VZV) has been implicated in Alzheimer’s disease (AD), and vaccination against shingles, caused by VZV, has been found to decrease the risk of AD/dementia. Previous work in our lab demonstrated that hiNSCs infected with VZV did not show the main AD characteristics, Abeta and P‐tau accumulation, which HSV‐1 does cause, but did show gliosis and increased levels of pro‐inflammatory cytokines, suggesting that VZV’s action relating to AD/dementia is indirect. Strikingly, we found that VZV infection of cells quiescently infected with HSV‐1 causes reactivation of HSV‐1 and consequent AD‐like changes, including Abeta and P‐tau accumulation.

**Methods:**

Given the established efficacy of vaccination against shingles in decreasing AD risk, we sought to determine whether neutralizing antibodies generated by vaccination, could attenuate the established effects of VZV in reactivating HSV‐1‐induced AD. We utilized hiNSCs latently infected with HSV‐1 and subsequently infected with VZV in the absence or presence of neutralizing VZV, then assessed expression of Abeta and P‐tau *in vitro*.

**Results:**

We found that neutralizing antibody against VZV attenuated VZV infection and thus reactivation of HSV‐1 and associated downstream AD‐like phenotypes in hiNSCs.

**Conclusions:**

These findings may provide mechanistic insight into the epidemiological evidence that VZV vaccination provides neuroprotection against AD/dementia.

